# Development of a Composite Measure of Product Adherence, Protocol Compliance, and Semen Exposure Using DNA and Protein Biomarkers for Topical HIV Prevention Studies

**DOI:** 10.1371/journal.pone.0114368

**Published:** 2014-12-09

**Authors:** Terry A. Jacot, Ashley Nelson, Andrea Thurman, Angela D. M. Kashuba, David F. Archer, Gustavo F. Doncel

**Affiliations:** 1 CONRAD, Department of Obstetrics and Gynecology, Eastern Virginia Medical School, Norfolk, Virginia, United States of America; 2 University of North Carolina Eshelman School of Pharmacy and University of North Carolina Center for AIDS Research, University of North Carolina, Chapel Hill, North Carolina, United States of America; Centers for Disease Control and Prevention, United States of America

## Abstract

**Background:**

Poor and inconsistent use of study products has hindered clinical HIV prevention studies. It is important to be able to monitor product adherence and protocol compliance in order to determine microbicide efficacy and safety more accurately. Current methods for monitoring adherence are subjective, non-specific, or invasive. Herein, we present a composite, objective measure of product adherence and protocol compliance to assess vaginal insertion, semen exposure and drug expulsion utilizing DNA, protein, and drug isolated directly from returned, vaginally used gel applicators.

**Methods:**

DNA, vaginal cells, and residual tenofovir were isolated from vaginally inserted applicators. Vaginal and semen biomarkers were amplified using a multiplex PCR to determine vaginal insertion. Vaginal cells were fixed followed by cytokeratin 4 immunocytochemistry to confirm DNA assessment of vaginal insertion. Tenofovir was extracted and quantitated through LC-MS/MS.

**Results:**

DNA isolated from vaginally inserted applicators were positive for vaginal bacteria DNA and the control eukaryotic gene, amelogenin, while manually handled, “sham”, applicators were negative for both. Semen exposure was independently determined by simultaneous amplification of one or both Y-chromosomal genes, SRY and TSPY4. Vaginal insertion determination by DNA analysis was further confirmed by positive cytokeratin 4 (CK4) immunocytochemistry of vaginal cells remaining on the gel applicators. On the contrary, sham applicators provided very few cells when swabbed, and they were all negative for CK4. CK4 was not found in epidermal cells from the hand. Drug expulsion was detected through quantitation of residual gel present on the surface of returned applicators. Sham applicators had no detectable tenofovir.

**Conclusion:**

Utilizing a composite, triple marker based panel of DNA, protein, and drug present on the surface of returned vaginal gel applicators, it is possible to determine, objectively and non-invasively, product adherence, protocol compliance, and semen exposure in microbicide trials.

## Introduction

More than 30 years into the epidemic, HIV infection remains a significant global public health issue, particularly in resource constrained countries [1]. Microbicides are products that can be applied vaginally to help reduce the risk of sexually transmitted infections (STIs), in particular HIV. This family of products includes antiretroviral-based microbicides, which have been developed with the intent to provide women with pre-exposure prophylaxis against HIV infection. While there are discordant results on reduced HIV incidence among various HIV prevention studies, protocol adherence data suggest that detection of drug in the participants was always associated with efficacy [2]. The CAPRISA 004 trial in South Africa investigating TFV 1% vaginal gel demonstrated an overall 39% reduction in HIV incidence by intent-to-treat analysis with higher degree of protection in women who used the product in more than 80% of the sex acts [3]. Furthermore, concentrations of tenofovir in cervicovaginal fluid in excess of 1000 ng/mL were associated with>70% protection [4], [5].

Poor adherence hinders interpretation of final data and leads to underestimation of drug efficacy. To further complicate this issue, there is ample literature demonstrating that self-report of compliance almost always overestimates protocol and product adherence [6]. Current measures of adherence include number of applicators collected [3], visual inspection of returned, used applicators (VIRA) [7], dye stain assays (DSA) using trypan blue [8] or FD&C Blue Dye No. 1 [9], [10], ultraviolet light illumination (UVL) of applicators [7], [11], or electronic monitoring such as the Wisebag [11], [12]. While all have been validated, each method is still limited by subjectivity or self-report on sexual activity. For example, DSA and UVL measures vaginal insertion by visual determination of positive staining or fluorescence of proteins in vaginal secretions. Subjectivity and different levels of training of the readers hinders consistent accuracy of adherence determination. The two tests are also not specific enough to determine semen exposure independent of vaginal insertion. The Wisebag monitors applicator use by electronically detecting when the bag is opened. This approach does not provide information on the number of applicators retrieved when the bag is opened. It also does not provide information as to whether applicators were retrieved around the time of coital activity. The Wisebag approach as well as counting returned applicators still depends upon self-report of sex acts in order to determine adherence. Finally, no current adherence measurement can determine whether vaginal insertion equates to vaginal gel explusion. To accurately determine appropriate gel applicator use as well as potential semen exposure, which reflects HIV exposure and risk of infection, we set out to identify objective, biological, and quantifiable markers of vaginal insertion, semen exposure, and drug expulsion that could be measured directly from used applicators with relative ease in a non-invasive manner.

For detection of vaginal insertion, we used vaginal bacterial DNA markers amplified by PCR. The human vagina has a well described microbiota that helps maintain a normal vaginal physiology and pH [13]. This microbiota has known fluctuations which can be used for diagnostic purposes [14]. Changes due to diseases like bacterial vaginosis have been detected through PCR amplification of bacterial DNA isolated from vaginal swabs [15]. Forensic reports have demonstrated the use of bacterial DNA for identification of vaginal fluids [16]–[18].

Although DNA is very stable and easily amplified, it is not always specific to a cell type or anatomic location. This also applies to bacterial DNA. It is well known, however, that differential expression of proteins can distinguish cellular phenotypes. A previous study reported that epithelial cells isolated from vaginal swabs expressed cytokeratin 4 (CK4) while those isolated from epidermal swabs did not [19]. We therefore used immunocytochemical detection of CK4 as a confirmatory marker of vaginal insertion. For semen detection, we previously developed a multiplex PCR system, which amplifies two Y-chomosomal genes, SRY (Sex-determining region in the Y chromosome) and TSPY4 (Testis-specific protein Y encoded 4), and an internal control gene, amelogenin. These male-specific genes can be detected in cervicovaginal swabs obtained from women up to 1 week post-semen exposure [20].

An additional requirement for correct product use is expulsion of product, once the applicator is inserted into the vagina around coital activity. TFV in human genital and colorectal tissue can be quantitated successfully by LC-MS/MS analysis [21], [22]. Therefore, we determined whether residual TFV gel from swabbed vaginally inserted applicators could be extracted and quantitated, as an indication of gel expulsion.

In this report, we present proof-of-concept data for detecting vaginal insertion and semen exposure using DNA and protein isolated from a single, vaginally inserted applicator. These markers were validated against “sham” (uninserted applicators) and epidermal cells from the hand. Completing the triple marker panel of adherence, we also present feasibility data in measuring residual TFV from the surface of vaginally inserted gel applicators.

## Materials and Methods

### Human subject research ethics statement

De-identified, returned, used gel applicators were obtained from women participating in two clinical trials utilizing either hydroxyethylcellulose (HEC) placebo gel (CONRAD D13-125, #NCT01804023) or TFV 1% gel (CONRAD A12-124, #NCT01810315) applicators. Both studies were approved by the Chesapeake Institutional Review Board (Pro 00008154) and Eastern Virginia Medical School (EVMS) Institutional Review Board (13-02-FB-0017), respectively. All women provided an informed, written consent for use of the samples. Semen was obtained with written, informed consent under a protocol approved by the EVMS Institutional Review Board (#13-02-FB-0031).

### Isolation of DNA and vaginal cells from vaginally inserted applicators

De-identified hydroxyethylcellulose (HEC) or “placebo” gel applicators were used for establishing the protocol to isolate DNA and vaginal cells on the applicator. The CONRAD D13-125 study for which these applicators were obtained [23] involved healthy HIV-negative women between the ages 18-50 whereby HEC gel applicators were vaginally inserted by the clinical investigator. Some applicators were inserted, removed, and HEC gel expelled into a waste container while some applicators were inserted and gel vaginally expelled before removal. Other applicators (“sham”) were manually handled without gloves by the participant. The sham applicators were prepared by taking the applicator out of the package, connecting the plunger, removing the cap, and expelling gel into a waste can rather than inserting into the vagina. The applicator was then placed back in the package.

**Figure 1:**
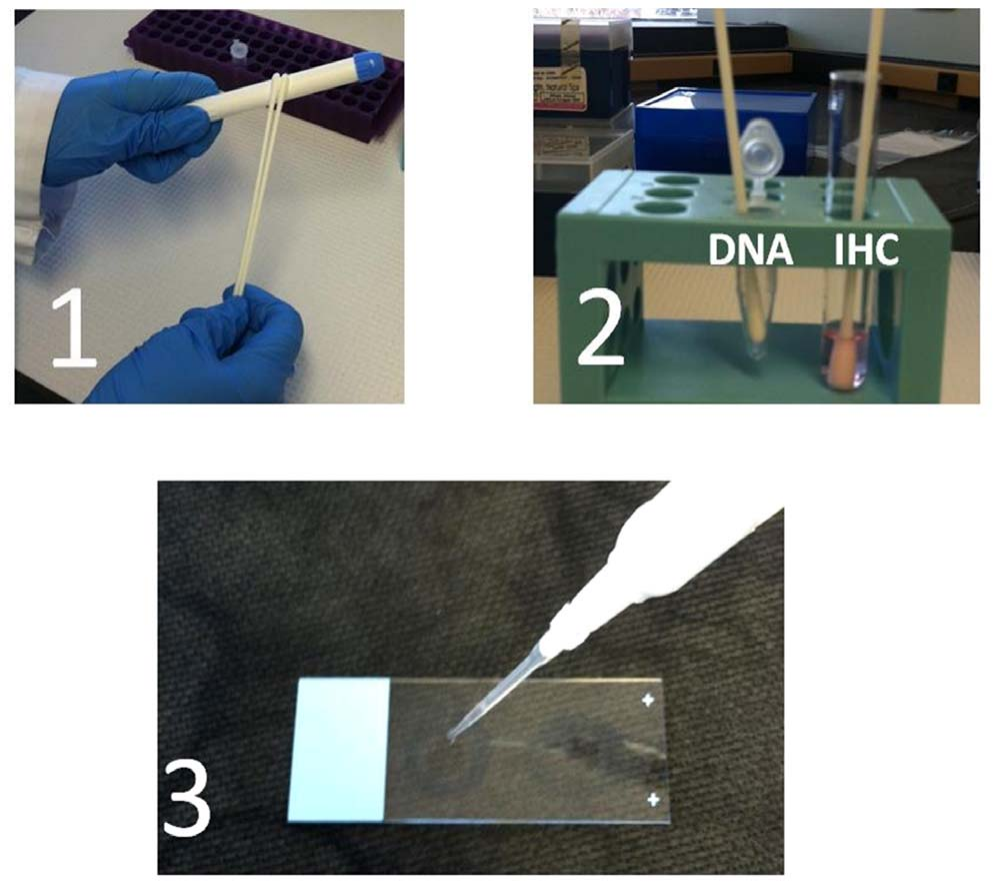
Isolation of DNA and Vaginal Cells from Inserted Gel Applicators. Vaginally inserted applicators or sham applicators were swabbed with a double headed rayon swab (1) to which one head was placed in a lysis buffer for DNA extraction and the other in Cytorich red for fixation of vaginal cells (2). The fixed vaginal cells were spotted onto slides for cytokeratin 4 immunohistochemistry (3).

In the research lab, applicators were swabbed with a double headed rayon Starplex swab (Starplex Scientific, Cleveland, TN) (Fig. 1). The swab heads were snapped apart, and one swab head was placed in a lysis buffer for DNA extraction as previously described [20]. Briefly, the swab head in the lysis buffer was incubated at 55°C for 1 hour before extraction using the Qiagen DNA Mini kit (Qiagen, Valencia CA). DNA was quantitated before using for PCR analysis. The other swab head was used for isolating vaginal epithelial cells by a modified, but previously described method [19]. The swabs were placed in 1 ml Cytorich Red solution (Thermo- Scientific, Kalamazoo, MI) for 5 minutes. Swabs were then removed and cell suspensions shaken for 10 minutes. The fixed cells were pelleted by spinning at 5000 rpm for 10 minutes and resuspended in 20 µL Cytorich Red. The cells were spotted onto slides at 5 µL and dried overnight for hematoxylin and CK4 immunocytochemistry.

### Vaginal–Semen multiplex PCR

The previously established multiplex PCR containing primers for Y-chromosomal genes was expanded to include various primers specific for seven bacterial species prevalent in vaginal microbiota. Using published sequences [15], [24]–[26], the primers shown in Table 1 were incorporated into the PCR protocol using the Qiagen Multiplex Plus PCR Kit (Qiagen, Valencia, CA). DNA from vaginal swabs, semen, or vaginally inserted gel applicators was used as template for the PCR. The slightly modified cycling protocol was as follows: One cycle of 95°C for 5 minutes, 35–40 cycles of 95°C for 30 seconds, 60°C for 90 seconds, 72°C for 60 seconds, and 1 cycle of 68°C for 10 minutes. After completion of the PCR cycles, the amplified DNA products were analyzed using the Agilent 2100 Bioanalyzer (Agilent Technologies, Santa Clara, CA) in conjunction with its DNA 1000 kit. PCR products were visualized by placing 1 µL of the PCR reaction to the DNA LabChip (Agilent Technologies, Santa Clara, CA) and placed in the 2100 Bioanalyzer, which provided the size and the concentration (ng/µL) of the amplified PCR products in the 50 µL PCR reaction.

**Table 1:** Vaginal Bacteria Primers for Multiplex PCR.

Species	PCR Product [reference]
*Lactobacillus gasseri*	162 bp [25]
*Prevotella buccalis-like*	192 bp [25]
*Megasphaera elsdenii-like (phylotype 1)*	211 bp [24]
*Eggerthella-like*	238 bp [15]
*BVAB1*	261 bp [15]
*Lactobacillus crispatus*	571 bp [24]
*Lactobacillus jensenii*	700 bp [26]

For testing the multiplex PCR using semen/sperm DNA as template, semen/sperm was obtained from healthy, normozoospermic, non-vasectomized donors under EVMS-approved IRB protocol #13-02-FB-003. The sperm DNA used in this study was extracted from a pooled sample of 3 donors. Semen DNA was extracted from 1 donor.

### Cytokeratin immunocytochemistry

Slides spotted with vaginal cells obtained from swabbed gel applicators were washed in 100%, 95%, and 70% ethanol sequentially, for 5 minutes each. After final washes in dH2O and PBS, slides were blocked with 3% BSA for 30 minutes before adding 1∶100 of a rabbit anti-human cytokeratin 4 primary antibody (Abcam, Cambridge, MA) or 1∶100 of a mouse anti-human cytokeratin 10 (Santa Cruz, Dallas, TX) in blocking buffer for 1 hour. An equivalent amount of normal rabbit or mouse IgG (Santa Cruz, Dallas, TX) was used as a negative control. After two washes in PBS, 1∶200 of a biotinylated goat anti-rabbit or goat anti-mouse IgG secondary antibody (Vector Laboratories, Burlingame, CA) was incubated for 30 minutes. Vectastain Elite ABC kit (Vector Laboratories, Burlingame, CA) in combination with AEC chromagen (ScyTek Laboratories, Logan, UT) was used for detection according to manufacturers' instructions. Cells were counterstained with hematoxylin before mounting in aqueous mounting media (DAKO, Carpinteria, CA) and coverslipping.

### Measurement of residual TFV on vaginally inserted applicators

TFV 1% gel applicators were obtained from an ongoing clinical study (CONRAD A12-124) whereby premenopausal women between 21–45 years, in their homes, vaginally inserted and expelled one gel applicator. Two hours later, they vaginally inserted and expelled a second applicator to simulate the BAT24 dosing regimen previously determined to successfully reduce HIV incidence in the CAPRISA 004 study [3]. To determine whether residual TFV on the external surface of the applicators could be measured, a third swab head was incorporated into the lab protocol described above. In this protocol, three swab heads were simultaneously used to swab the surface of the applicator. While the original two heads were used for DNA/CK4 biomarkers, the newly added swab head was frozen at −80°C until ready for LC-MS/MS analysis of TFV. Quantification of tenofovir was performed by extracting the drug from calibration standards, quality control samples, and study samples using protein precipitation and a stable labeled tenofovir as internal standard. Tenofovir on swabs were solvated in 2mL, and 100 µL aliquots were utilized for analysis. Tenofovir was eluted from a Waters Atlantis T3 (100x2, 1 mm, 3micron) analytical column with mobile phases consisting of formic acid, acetonitrile and water, detected on an AB Sciex API-5000 triple quadrupole mass spectrometer, and data collected with Sciex Analyst Chromatography Software. The calibration range was 0.1 ng to 1,000 ng of tenofovir per swab, with intra- and interday variability and precision of <15% and <10%, respectively.

## Results

### Vaginal biomarkers-semen biomarkers multiplex PCR

**Figure 2:**
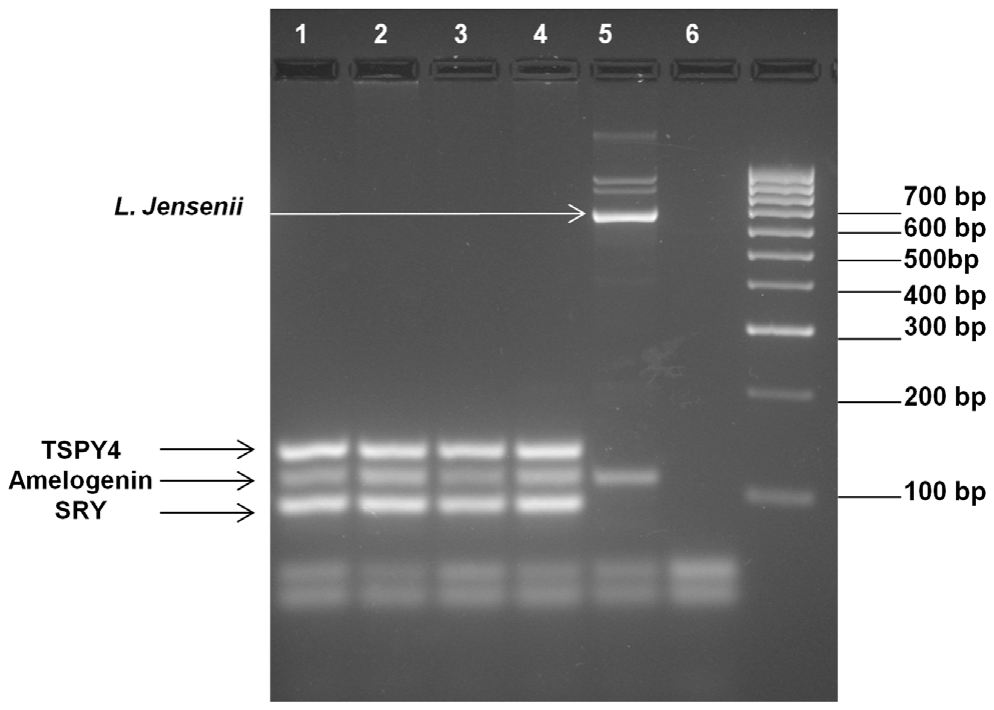
Lack of Bacterial Marker DNA Amplification in Semen or Sperm DNA. 30 and 90 ng, respectively of sperm DNA (lanes 1–2) and semen DNA (lanes 3–4) were used as template DNA for the expanded multiplex PCR. No bacterial markers were amplified. Only the Y-chromosomal DNA markers, TSPY4 and SRY, along with the internal control gene, amelogenin, were amplified. Vaginal swab DNA showing *L. jensenii* amplification was used as a positive control (lane 5). Lane 6 is a PCR negative control.

**Figure 3:**
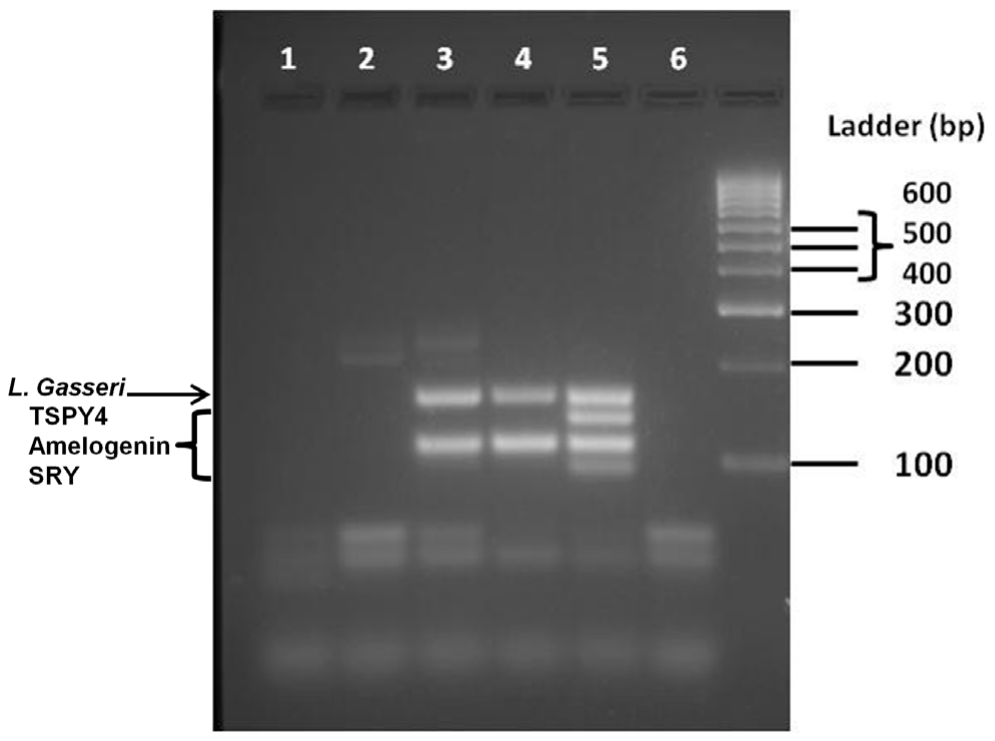
Representative Multiplex PCR Amplification of DNA Biomarkers from Vaginally Inserted and Sham Applicators. Identical amounts of template DNA from sham (lane 2) and vaginally inserted (lane 3 and 5) HEC gel applicators obtained from one woman were used for the multiplex PCR. Vaginal swab DNA from the same woman was used as a positive control (lane 4). A control applicator which was simply taken out of the package and swabbed was also compared as a negative control (lane 1). A PCR negative control is included (lane 6). *L. gasseri* was amplified in only the vaginally inserted applicator DNA and positive control vaginal swab DNA. Semen exposure is detected as TSPY4 and SRY gene amplification (lane 5). No amelogenin amplification is seen in the control or sham applicator (lane 1–2).

**Figure 4:**
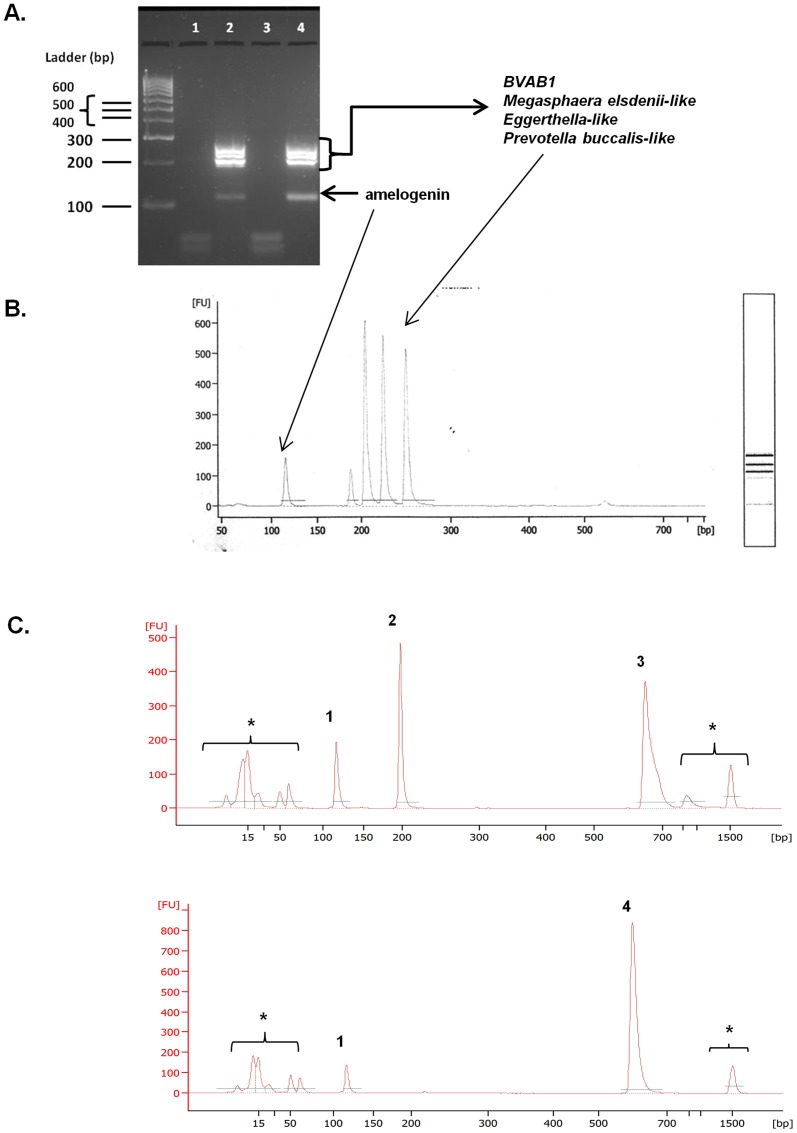
DNA Biomarker Panel Applies to Ethnically Diverse Populations. A) Agarose gel electrophoresis analysis showing multiplex PCR amplification of identical amounts of template DNA from sham (lane 1) and vaginally inserted (lane 2) applicators from an African-American woman. Four bacterial markers listed above plus amelogenin were only amplified in the DNA from a vaginally inserted HEC gel applicator and not in DNA from a sham applicator. These bacterial markers were identical to those amplified in the corresponding vaginal swab DNA (Lane 4). A PCR negative control is also included (lane 3). B) PCR analysis using the Bioanalyzer 2100 confirms the 4 bacterial markers and amelogenin. C) Electropherograms of DNA biomarkers from vaginally inserted applicators from two additional African-American women demonstrating different panels of bacterial DNA markers. Peak #1 is the control gene amelogenin, peak #2 is *Prevotella buccalis-like*, peak #3 is *L. jensenii*, and peak #4 is *L. crispatus*. *Non-specific primer dimers and DNA sizing markers.

DNA isolated from vaginally inserted HEC gel applicators without vaginal gel expulsion was analyzed for bacterial and semen biomarkers. Using our previously developed semen exposure multiplex PCR, the technique was expanded to include detection of vaginal bacteria using published primer sequences (Table 1). The panel of vaginal bacterial species encompasses lactobacilli since this species is highly prevalent in the vagina. Also included are non-lactobacilli which have been reported in women with BV [15], but also in healthy women of non-Caucasian ethnicities [13]. The chosen bacterial species needed to be undetectable in semen/sperm DNA. This was a necessary criterion in order to specifically distinguish vaginal insertion of the gel applicator from semen exposure, two independent events. The final multiplex PCR, tested against semen/sperm DNA, confirmed negative amplification of bacterial markers (Fig. 2). In these samples, the only products amplified were the human control gene, amelogenin, and Y-chromosomal genes, TSPY4 and SRY. The multiplex PCR was able to detect bacterial DNA present on vaginally inserted gel applicators with or without semen exposure (Fig. 3, Lanes 3, 5). Unlike DNA from vaginally inserted applicators, no bacterial PCR products were amplified from an ungloved, manually handled, un-inserted applicator (sham, Lane 2). It is important to note that amelogenin was not amplified in DNA from a sham applicator suggesting that there were not enough epidermal cells from the hand transferred to the applicator for amplification of the gene in this PCR platform. Therefore, amplification of amelogenin and bacterial marker(s) together is necessary to conclude that the applicator was vaginally inserted. While Fig. 3 shows one bacterial species present on the applicator, the primers in the multiplex PCR were chosen to potentially detect different patterns of bacterial species found in different women of different races. Fig. 4A is an example of DNA from a vaginally inserted applicator of an African-American woman demonstrating amplification of 4 bacterial markers. An electropherogram of the amplified bacterial markers generated by the Bioanalyzer 2100 is also included (Fig. 4B). Because of the higher resolution of the individual PCR products, the Bioanalyzer was chosen to be the method of choice for PCR analysis of future studies. Fig. 4C are electropherograms from applicator DNA of two more African American women showing amplification of amelogenin plus differing patterns of bacterial markers.

### Cytokeratin 4 as a marker of cervicovaginal cells

**Figure 5:**
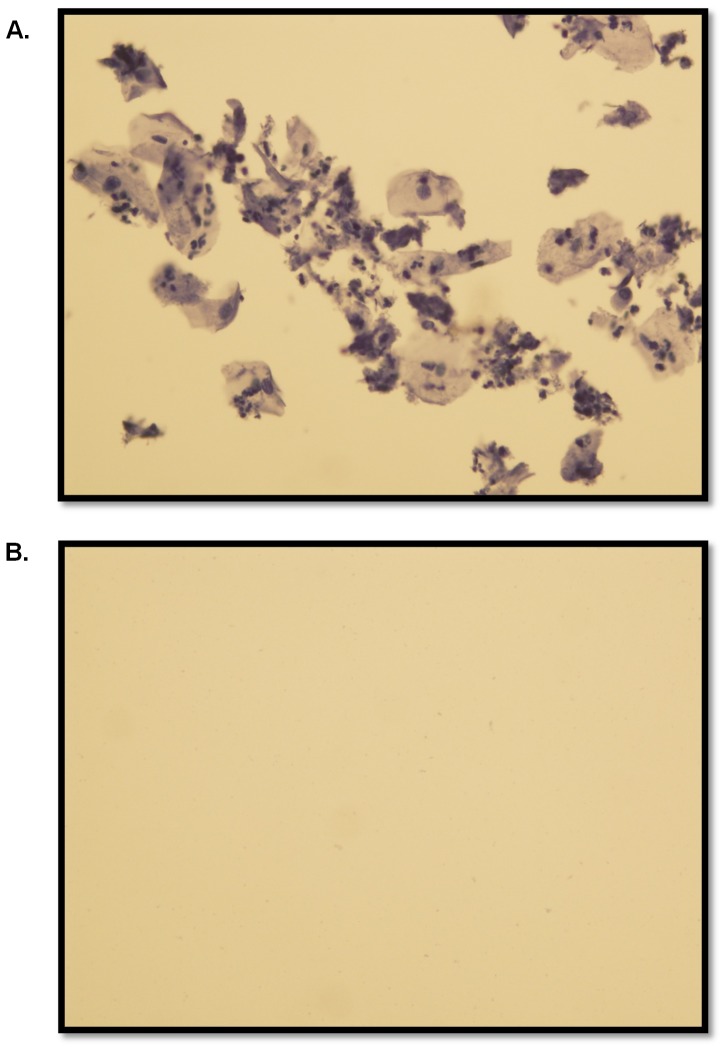
Hematoxylin Stain Discriminates Vaginally Inserted from Sham Applicators. Swabbed cells from applicators were isolated and fixed in Cytorich Red solution. 5 µl of the resulting 20 µl suspensions from vaginally inserted applicators (A) and sham applicators (B) were spotted and dried overnight for staining the next day. The number of cells transferred from the hand to applicator during handling is too low to detect cells in a 5 µl aliquot (B).

**Figure 6:**
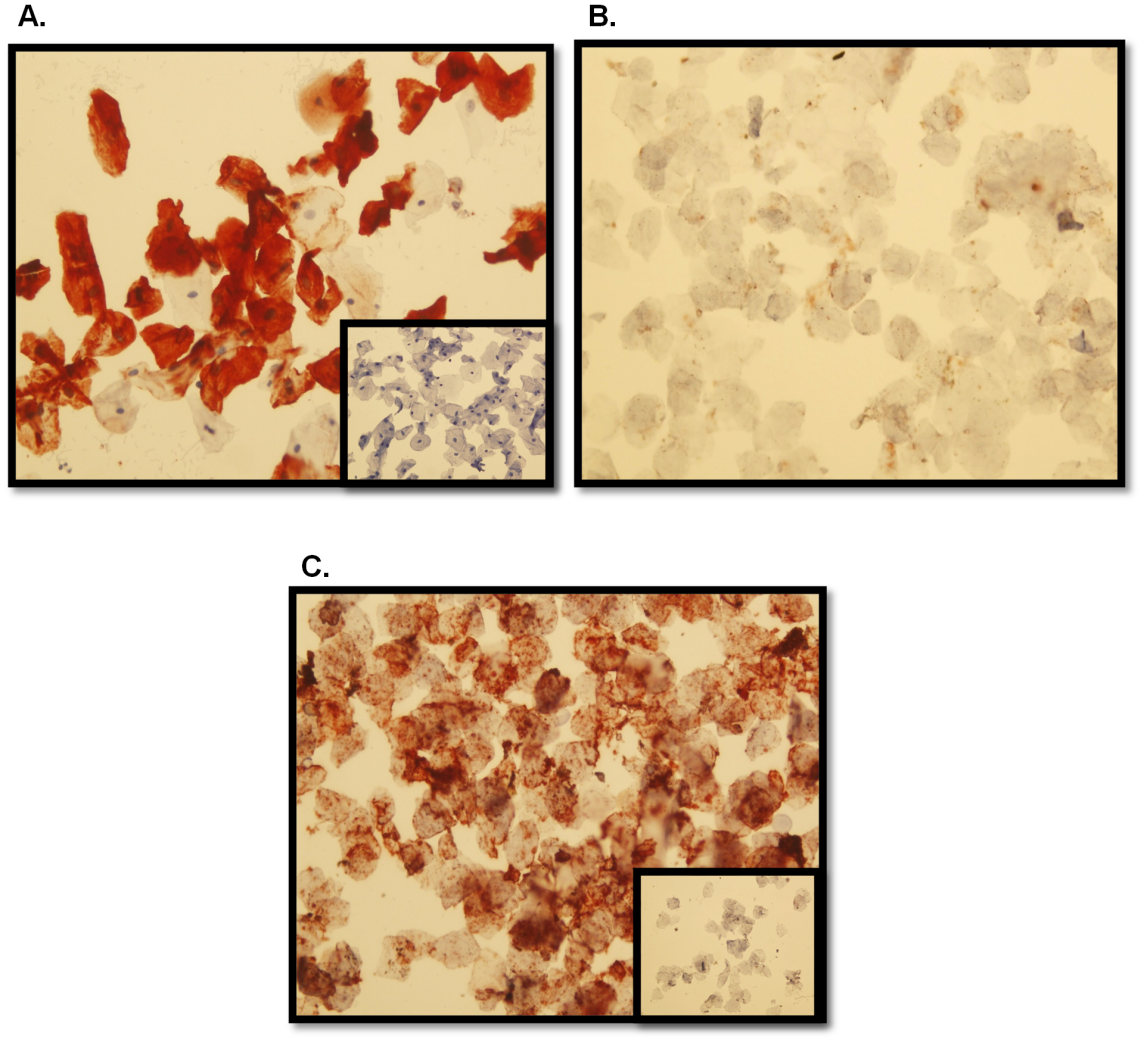
Representative Cytokeratin 4 (CK4) Immunocytochemistry in Cells From Vaginally Inserted Applicators and Hand Swabs. Cells from vaginally inserted HEC gel applicators were positive for CK4 (A) while epidermal cells were negative (B). To confirm the quality of the epidermal cells, CK10 expression was determined as a positive control (C); Normal rabbit or mouse IgG negative controls (inset pictures).

While protein biomarkers are not as sensitive as DNA biomarkers, they offer high specificity. As part of this composite measure of gel applicator adherence, cells were also isolated from vaginally inserted HEC gel applicators described above. A hematoxylin stain of the cells was done to confirm the feasibility of the method for isolating the cells from the applicator, and the result was compared to a swabbed sham applicator. There was a clear difference in cell numbers recovered from vaginally inserted and sham applicators (Fig. 5). Sham applicators do not provide the abundance of cells, if any, that vaginally inserted applicators provide. To further confirm that the hematoxylin stained cells were vaginal in origin, the presence of CK4 was determined using immunocytochemistry. Published data has demonstrated the presence of CK4 in cells from vaginal swabs, but not from epidermal swabs [19]. As predicted vaginal cells recovered from an inserted applicator were positive for CK4 while epidermal cells from the hand were not (Fig. 6A, 6B). Cytokeratin 10 (CK10) was detected in the epidermal cells as a positive control confirming the validity of the negative CK4 result (Fig. 6C). CK4 expression in the recovered cells is an additional and specific confirmation that the gel applicator was vaginally inserted.

### Detection of DNA/protein biomarkers on stored applicators and upon exposure to vaginal HEC gel expulsion

**Figure 7:**
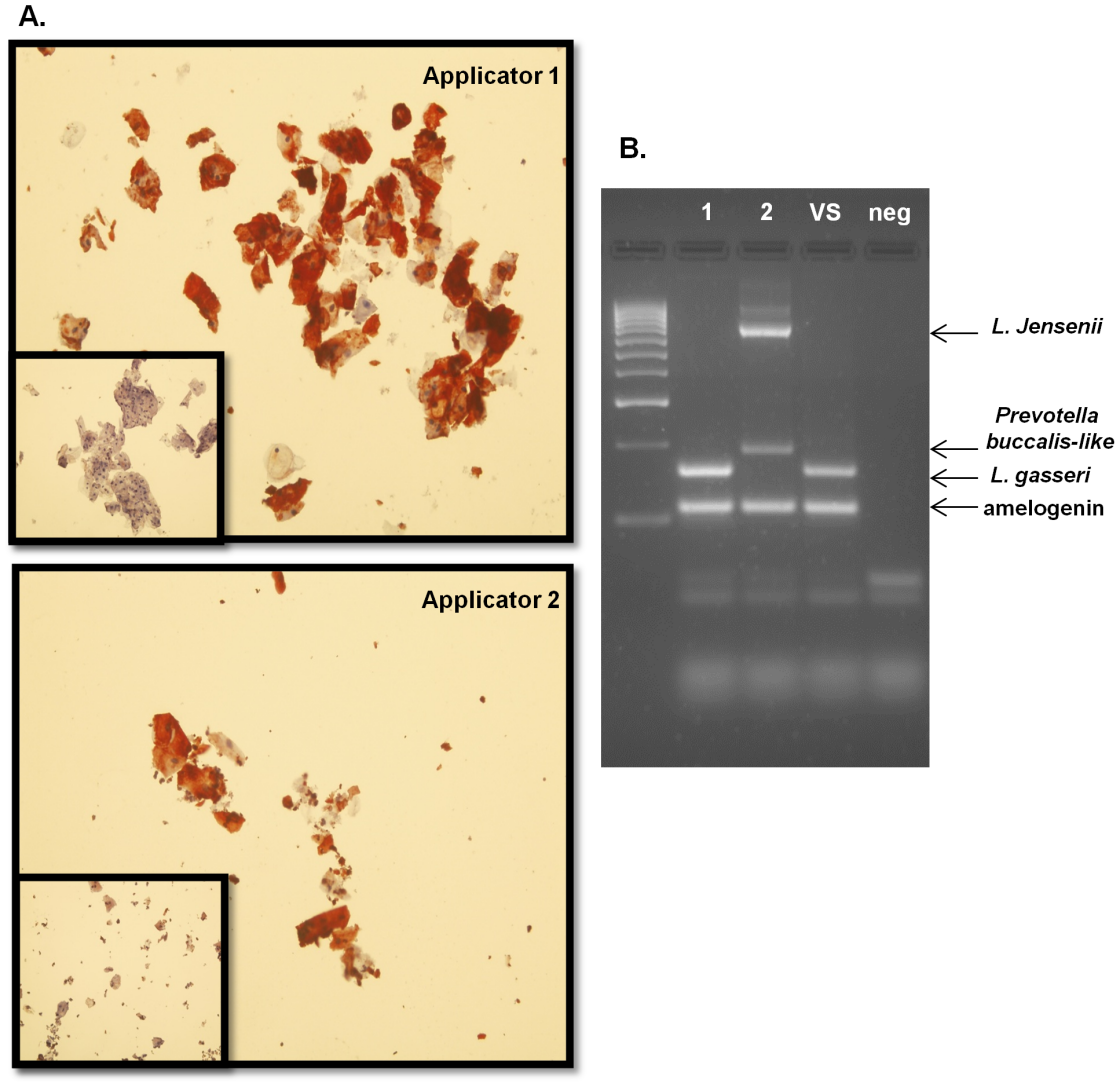
DNA and CK4 Biomarkers on Applicators Stored for 30 days. After 30 days of storage at room temperature, applicators from two women (#1 and #2) were swabbed and DNA/cells isolated. CK4 expression was positive (A) while amelogenin and bacterial markers were amplified by the multiplex PCR (B) confirming stability of the biomarkers over the course of 30 days. Vaginal swab DNA from woman #1(“VS”) is included as a positive control to confirm that identical amplified bacterial species were found on the applicator #1 (Lane 1). A no DNA PCR control is also included (“neg”); Normal rabbit IgG negative control (inset pictures).

Logistically, it is not practical to have participants in a microbicide gel applicator study return used applicators immediately. Therefore, we determined whether the DNA and CK4 biomarkers could be detected from vaginally inserted applicators after storage in the original wrappers for 30 days at room temperature. As shown in Fig. 7, vaginal CK4 protein (A) and vaginal bacterial DNA markers (B) were successfully detected from applicators from two women (#1, 2) stored for 30 days. Vaginal swab DNA from one of the women (“VS”) was included as a positive control to show that the bacterial species amplified from the stored applicator was identical to a vaginal swab from that same woman (#1).

**Figure 8:**
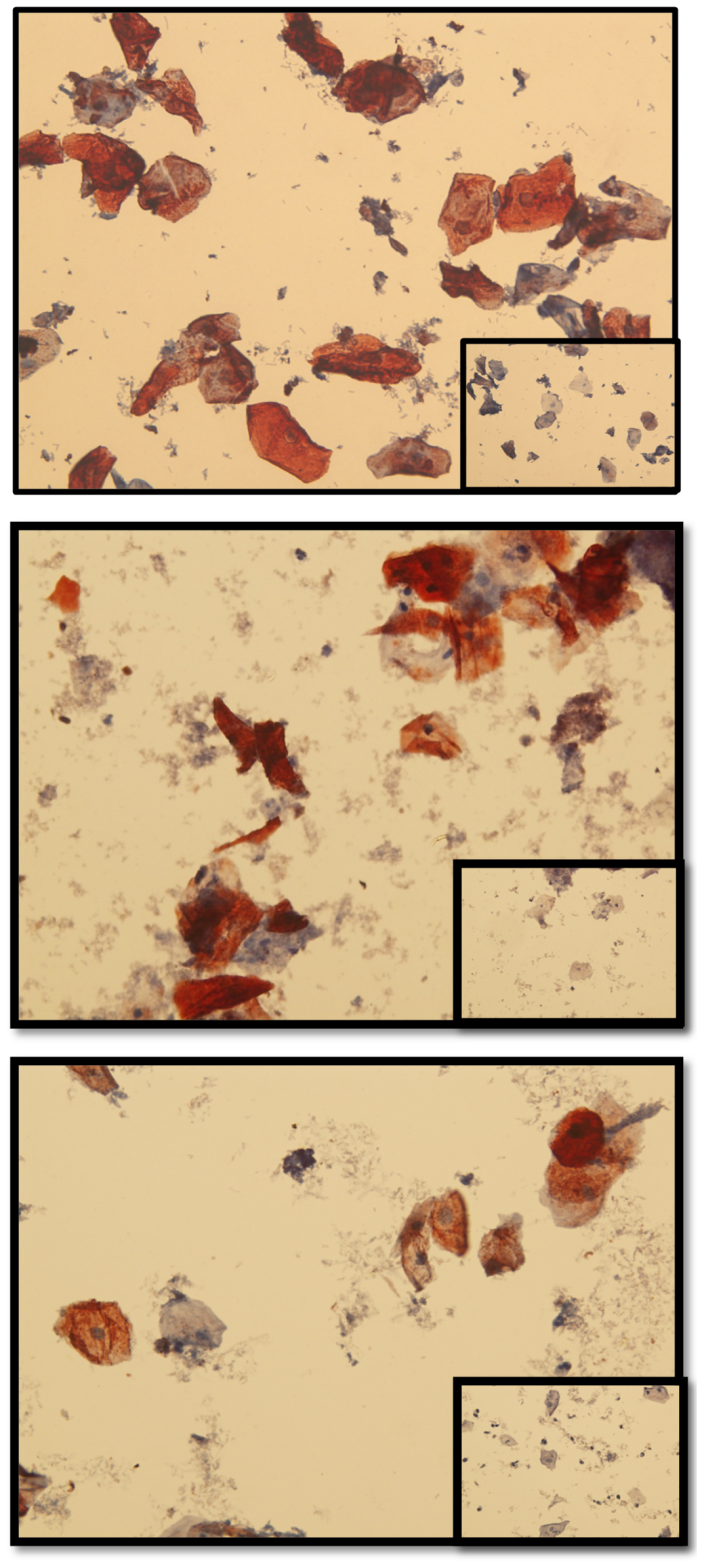
Hydroxyethylcellulose (HEC) Placebo Gel Does Not Interfere with CK4 Biomarker Detection. HEC gel applicators were inserted and gel expelled into the vaginas of three different women. The applicators were swabbed for analysis of CK4 expression in isolated vaginal cells. While the observed particles above appear to be associated with the presence of gel, the cells from all three women still demonstrated positive CK4 expression. Normal rabbit IgG negative control (inset pictures).

**Figure 9:**
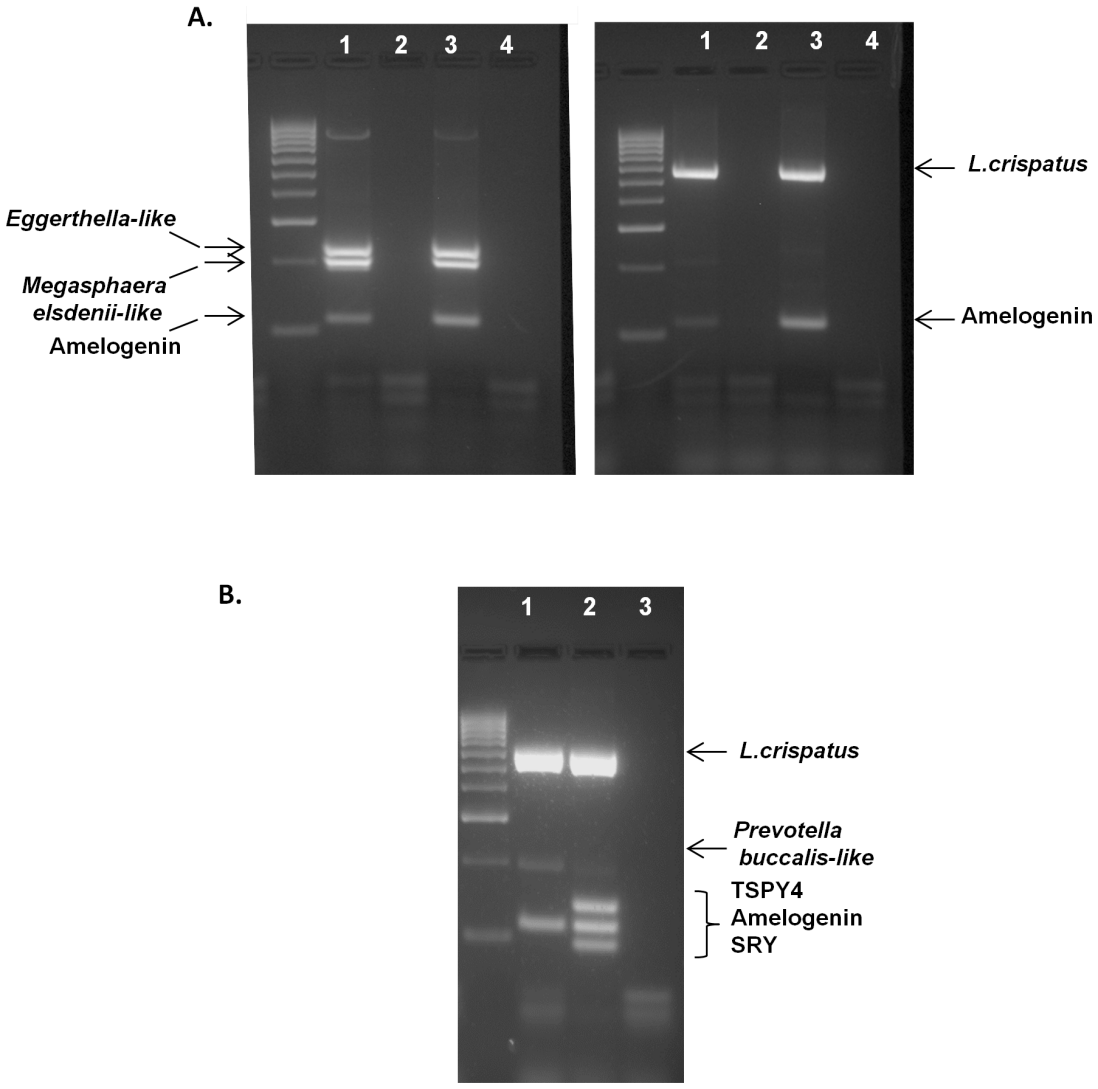
Hydroxyethylcellulose (HEC) Placebo Gel Does Not Interfere with DNA Biomarker Detection. A) Different patterns of DNA biomarkers were still detected in the presence of vaginally expelled HEC gel (Lane 1). These patterns matched the corresponding patterns observed from vaginal swab DNA of those same women (Lane 3). DNA from sham applicators manually handled by the women (Lane 2) and No DNA PCR (Lane 4) controls were negative. B) Exposure of HEC gel did not interfere with the DNA biomarkers before (Lane 1) or after semen exposure (Lane 2). No DNA PCR negative control is included (Lane 3).

While the data shown above confirms that biomarkers of vaginal insertion and semen exposure can be isolated from HEC gel applicators that were inserted without gel expulsion, it was necessary to determine if vaginally expelled HEC gel interferes with biomarker detection. Fig. 8 shows cells from three different women displaying positive CK4 detection when HEC gel was vaginally expelled. Fig. 9A shows different examples of DNA biomarkers isolated from vaginally inserted and gel expelled applicators. The pattern of bacterial biomarkers plus amelogenin from the applicators (Lane 1) was the same as those observed from vaginal swab DNA of the same woman (Lane 3). Sham applicators handled by the women were negative for those markers, including amelogenin (Lane 2). In addition, semen DNA biomarkers were not affected by HEC gel (Fig. 9B).

### Measurement of Residual TFV and Biomarkers on Vaginally Inserted Applicators

**Figure 10:**
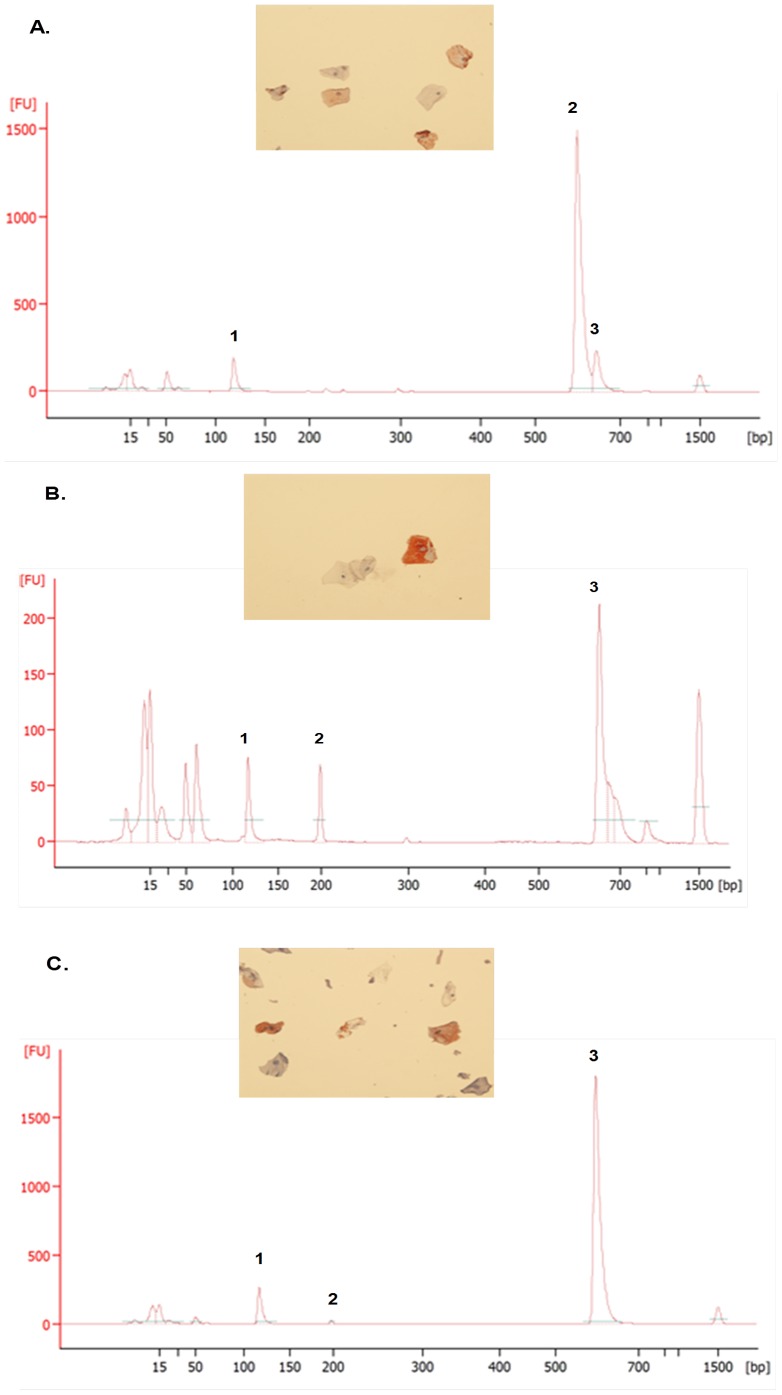
TFV 1% Gel Does Not Interfere with DNA and CK4 Biomarker Detection. Examples of 3 different women that vaginally inserted and expelled TFV 1% gel applicators before returning the applicators to the clinic. Applicators were stored in the clinic for 6 days (A) or as long as 7 months (B, C) before swabbing for DNA and CK4 biomarkers. All showed evidence of vaginal insertion by amplification of amelogenin (peak #1) plus bacterial markers and positive CK4 detection. The bacterial species detected are as follows: (A) #2, *L. crispatus*; #3, *L. jensenii*; (B) #2 *Prevotella buccalis-like*; #3 *L. jensenii*; (C) #2, *Prevotella buccalis-like*; #3, *L.crispatus*. The peak representing *Prevotella buccalis-like appears* very small because the Y-axis scale had to accommodate the abundant levels of *L.crispatus*.

The feasibility of measuring residual TFV remaining on applicators in which gel had been discharged vaginally was also determined. TFV gel applicators, vaginally inserted and discharged by four women, were swabbed, and those swabs extracted for TFV. These results were compared to sham applicators and unopened, unused applicators (“blanks”). Table 2 demonstrates the results of the LC-MS/MS analysis of TFV in those swabs. Swabs from all four vaginally inserted gel applicators (#1-4) had detectable TFV. Negative controls including sham and unused applicators (“blanks”) demonstrated no background TFV detection. Also included in the table are the storage times of each of the applicators. Applicators were stored at room temperature until ready for swabbing and subsequent drug analysis. The data clearly show that residual surface TFV is detectable on vaginally inserted applicators stored up to 55 days. This data confirms feasibility to measure drug expulsion simultaneously with biomarkers of vaginal insertion and semen exposure. Like HEC, TFV did not interfere in detecting biomarkers of vaginal insertion and semen exposure. Unlike the used HEC applicators described above which were vaginally inserted and gel expelled by the clinical investigator, the biomarker data shown in Fig. 10 are from TFV gel applicators that were vaginally inserted by three participants. The data also shows that DNA and CK4 biomarkers can be detected whether the applicator was stored for 6 days (A) or 7 months (B, C) at room temperature before being swabbed for analysis.

**Table 2:** Quantitation of Residual Tenofovir (TFV) on Gel Applicators.

	Vaginally Inserted (#1-4)				Controls	
	1	2	3	4	Sham^1^	Blank^2^
TFV (ng)	1120	1250	659	577	BLD^3^	BLD
Storage (days)	32	55	33	13	-	-

1 Manually handled, gel expelled in trash, n = 2.

2 Unopened applicator, removed from foil packet only, n = 2.

3 BLD  =  Below level of detection.

## Discussion

Confirming product adherence and protocol compliance in microbicide HIV prevention studies is necessary to rationally interpret the safety and efficacy of microbicides and other HIV prevention products [2]. In addition, a common requirement of participants in these types of studies is the use of condoms during coital activity to prevent semen exposure, an indicator of increased HIV risk. This report presents a composite, qualitative measurement of vaginal insertion (protocol compliance), semen exposure (HIV risk), and drug expulsion (product adherence) using objective biomarkers and residual drug present on vaginally inserted gel applicators. Vaginal gel applicators were chosen to develop this composite measure because, thus far, vaginal TFV 1% gel used before and after coitus is the only microbicide proven to reduce the risk of HIV acquisition in women [3]. Furthermore, this gel delivered by vaginal applicators is currently being tested in a phase III safety and effectiveness study (FACTS001) and an open-label study (CAPRISA008). The outcomes of these studies are clearly dependent on adherence and protocol compliance.

We previously reported the development of a multiplex PCR system to assess semen exposure by detecting the presence of TSPY4 and SRY, two y-chromosomal genes [20]. Using this system as a platform, DNA biomarkers were pursued to assess vaginal insertion of gel applicators. Similar to the Y-chromosome which is DNA specific only to the male gender, vaginal bacteria were used as targets for biomarkers since it could be distinguished from the ubiquitous human (female) DNA. There is ample literature describing fluctuations in the normal vaginal microbiome, particularly BV, using PCR or next generation sequencing [13], [15], which establishes that Lactobacilli are the predominant bacteria in the vaginas of premenopausal women. In addition, bacterial 16S rRNA sequences have been investigated for identification of body fluids of forensic biological samples [16], [17]. Therefore, we incorporated lactobacilli primers into our semen multiplex PCR. However, the PCR must have enough markers to identify a variety of women of different ethnicities. Studies comparing vaginal microbiomes of various ethnic groups have demonstrated that some groups had lower levels of lactobacilli suggesting that a normal vaginal flora may not be as lactobacilli dominant as previously believed [13], [14], [27]. Therefore, our multiplex PCR incorporates non-lactobacillus species as well. The applicator data of three African-American women presented in this report demonstrate the feasibility that the current panel of vaginal bacteria chosen should be able to detect vaginal bacteria from women of different races and ethnicities. This has been confirmed in a recently completed clinical study (CONRAD D13-125), whereby bacterial DNA biomarkers were detected on gel applicators from all 40 women, which encompassed Caucasian and African-American races as well as Hispanic ethnicity [23]. It also confirms that, for some women, non-lactobacillus species may predominate over lactobacilli. Therefore, inclusion of both lactobacillus and non-lactobacillus species is a necessary criterion for analyzing DNA-based adherence markers on vaginally inserted applicators from larger, more diverse cohorts of women. Since the data presented here represents women in the US, we recently started a study investigating these bacterial biomarkers on a subset of gel applicators used by African women who participated in the FACTS 001 study. The data generated from this study as well as future studies currently being planned will confirm the applicability of these biomarkers to different vaginal flora observed across diverse populations of women.

Combining vaginal bacterial markers with Y-chromosomal markers allows simultaneous detection of vaginal insertion of the applicator and possible semen exposure. Some of the current methods used to determine vaginal insertion of gel applicators employ visualization of vaginal fluid either by the naked eye under ambient light (VIRA) [7], dyes such as trypan blue [9], or ultraviolet light (UVL) fluorescence [7]. A major limitation of these methodologies is that the assessment is affected by the subjectivity of the reader. While trypan blue and UVL have better sensitivity than VIRA, they are not specific enough to distinguish between vaginal and other fluids. It is also well known that semen, and even other biological fluids fluoresce under UVL [28]. The particular plastic of the gel applicator being used affects DSA methodology [29]. Sensitivity and specificity was lower for HTI polypropylene plastic gel applicators versus polyethylene gel applicators. The data presented in this report utilized HTI polypropylene gel applicators. Comparing DNA and protein biomarkers with VIRA and UVL demonstrated that the overall biomarker sensitivity and specificity of 98% and 100%, respectively, was not hindered by this type of applicators [23].

While DNA biomarkers provide high sensitivity, they do not always provide high specificity as to cellular location of the biological sample. Cytokeratins are cytoskeletal components commonly investigated for identification of not just origin, but location of a biological sample [19]. Based on results of this previous report, we investigated the utility of cytokeratin 4 to confirm vaginal insertion versus manual handling (“sham”) of gel applicators. Our data agrees with the previous report that vaginal cells on an applicator express cytokeratin 4 while epidermal cells do not. Skin cells, as previously shown, are positive for cytokeratin 10. Vaginal cells isolated from the applicator are also positive to cytokeratin 10 to various degrees among different women. Slight positivity of CK10 in cells from vaginal swabs has also been observed so using CK10 for determining vaginal versus epidermal origin is less useful [19]. The present analysis excludes the possibility of false positives based on epidermal cells shed upon manual handling of the gel applicator by overall cell abundance (hematoxylin stained slide) and the presence or absence of cytokeratin 4. The very low cell yield from a manually handled “sham” applicator correlates with the absence of amelogenin gene amplification in sham applicator DNA. This observation, recently confirmed in a larger study [23], suggests that the chance for false positives generated from epidermal cells appears low. Therefore, even when contamination of the hand with vaginal bacteria is possible, amelogenin plus vaginal bacteria amplification together is indicative of vaginal insertion. Very low sample yield from the hand is not a rare event. This type of sample has been termed by the forensics field as “Touch DNA”, which is DNA isolated from shed skin cells transferred between two people or between an object and person during some type of physical contact. Because of the limited amount of sample present with this type of contact, there is still much ongoing investigation in being able to simultaneously determine not just a DNA profile but origin of the forensic sample [30].

The CK4 data presented in this report show variable yields of cells isolated from vaginally inserted applicators stored at different time points ranging from 6 days up to 7 months. Data in Fig. 10 plus unpublished observations do not demonstrate a strong correlation between cell yield and time of applicator storage. Current ongoing work suggests that variability in cell yield originates from the differences in the amount of vaginal cells shed from one woman to the next. Therefore, there is a possibility that cells shed and left on the used applicator may be too low to detect by hematoxylin stain or CK4 immunocytochemistry (false negatives). We believe DNA biomarkers should be able to overcome this limitation because the stability of DNA is much higher than protein. Because of this, additional experiments are being performed to adjust the laboratory protocols for obtaining optimal sample yields from various levels of vaginal material left of the applicators.

The “BAT 24” dosing regimen of TFV that has shown efficacy in reducing HIV acquisition in women involves a pre-coital and post-coital dose of TFV gel [3]. In this report, the presence of TFV or HEC gel did not affect the amplification of DNA biomarkers or the detection of CK4 in vaginal cells. The applicators could also be stored up to 30 days without loss of the DNA and protein biomarkers, and this timeframe has been successfully confirmed [23]. Preliminary data from applicators stored 7 months under climate controlled laboratory conditions suggest the possibility of even longer storages (Fig. 10). These results confirm the stability of DNA and CK4 biomarkers in determining vaginal insertion of applicators and semen exposure. Since Y-STR DNA typing has been successfully done with vaginal swabs stored for 25 years [31], we are planning to investigate the stability of the biomarkers upon longer storage times such as three and six months. These investigations include planning of future clinical studies in Africa to test the stability of the biomarkers on vaginally inserted applicators stored at different time points under field conditions of high heat and humidity. This is particularly important for CK4 analysis since proteins are less stable. The feasibility of adding a third swab to the current protocol for measuring residual TFV concentrations on vaginally inserted applicators was tested. Our results from vaginally inserted applicators for which TFV gel was expelled confirms that residual TFV swabbed off of the applicators stored up to 55 days can be detected by LC-MS/MS techniques. In addition, TFV was not detected on sham applicators suggesting that vaginal drug expulsion can be distinguished from sham applicators. This initial data has led to current, ongoing investigation to determine whether this approach can determine pre-coital versus post-coital gel expulsion.

This biomarker approach for determining adherence has been specifically designed for an applicator type of drug/gel delivery. It is still yet to be determined whether different aspects of the approach can be utilized for other delivery systems. This question is currently under intense investigation. Another requirement for achieving the most accurate interpretation of the data is the necessity that clinical studies utilizing this approach incorporate a vaginal swab positive control into the design. This requirement is important because the environment is an obvious source of bacteria. This potential source of external contamination plus the high sensitivity of the PCR technology increase chances that some amplified bacterial markers may not originate from the vagina. However, DNA isolated from the vaginal swab would confirm which species originated from the vagina. This is also necessary for DNA biomarkers alone to determine vaginal insertion when yields of isolated vaginal cells are too low or of low quality to confirm vaginal origin via CK4 immunocytochemistry. While the data presented here and our completed pilot study [23] demonstrate very good ability to distinguish sham handling versus vaginal insertion, the composite measure does not provide time-specific information of applicator use. In other words, we cannot definitely determine that the participant utilized the gel according to the BAT24 protocol versus feigning adherence by potentially inserting all applicators at one time point before returning to the clinic. Completing the development and validation of drug expulsion through LC-MS/MS will help provide an answer to this question. As it currently stands, this qualitative assessment of adherence objectively determines that applicators were vaginally inserted without total dependency on self-reports of sex acts and with less subjectivity of data interpretation.

In summary, we present an objective approach using DNA and protein biomarkers plus drug detection to determine vaginal insertion of gel applicators, semen exposure, and drug expulsion from a single returned applicator. Consistent and correct use of microbicides is needed to provide appropriate protection from HIV transmission [32]. Therefore, biomarkers of adherence are extremely desirable because data shows that subjective markers of adherence are unreliable. It is clear that some of the recent large phase III trials of PrEP products have failed to show product effectiveness due to low adherence [33], [34] because only a low percentage of blood samples showed detectable drug levels. It is therefore possible that many of the previous topical microbicides trials, which failed to show product effectiveness but for which we do not have objective markers of adherence, may have suffered from the same problem [35]–[37]. Solid adherence data utilizing more objective measures would enable the implementation of alternative trial designs based on high-adherence population enrichment.

Various delivery systems of microbicides are being developed and tested because women vary in their preferences, social context and sexual behavior, and these factors affect product adherence and effectiveness [38]. Future studies expanding the use of DNA and protein biomarkers of adherence can also aid in confirming subjective, self-reports of high or low acceptability to eventually develop better products that will be used successfully for HIV protection.
